# Different Immune Reconstitution between Cord Blood and Unrelated Bone Marrow Transplantation with Relation to Chronic Graft-versus-Host Disease

**Published:** 2020-01-01

**Authors:** Hitoshi Yoshida, Midori Koike, Yuma Tada, Keiichi Nakata, Akihisa Hino, Shigeo Fuji, Hiroaki Masaie, Chihiro Oka, Akemi Higeno, Atsushi Idota, Tomoyuki Yamasaki, Jun Ishikawa

**Affiliations:** 1 Department of Hematology, Osaka International Cancer Institute, Osaka, Japan; 2 Department of Laboratory, Osaka International Cancer Institute, Osaka, Japan

**Keywords:** Immune reconstitution, Cord blood transplantation, Unrelated bone marrow transplantation, Chronic GVHD

## Abstract

**Background:** Advances of allogeneic hematopoietic cell transplantation (allo-HCT) have brought long-term survival to the patients with hematologic malignancies. Chronic graft-versus-host disease (GVHD) is one of major problems for the long- term survivors after allo-HCT. Dysregulation of immune reconstitution has been reported to be involved in the pathogenesis of chronic GVHD. Differences of immune reconstitution between cord blood transplantation (CBT) and unrelated bone marrow transplantation (UBMT) remain unclear in long-term survivors. We investigated immune reconstitution in patients surviving for more than 2 years after CBT (n=21) or UBMT (n=20) without relapse of underlying disease.

**Materials and Methods:** Using flow cytometric analysis of peripheral blood, we investigated immune reconstitution of T cells, B cells, and NK cells between CBT and UBMT patients. We collected clinical data regarding allo-HCT and examined the relation of immune reconstitution to the development of chronic GVHD.

**Results:** Between CBT and UBMT patients, we found significant differences in absolute cell number of CD8+ as well as CD19+ cell and CD4/CD8 ratio even more than 2 years after allo-HCT. Among UBMT patients, absolute cell number of naive CD4+ cell was significantly lower in patients with chronic GVHD. In addition, we found significant differences in absolute cell number of CD19+ cell, especially naive B cell between patients with and without chronic GVHD in both CBT and UBMT patients.

**Conclusion:** These results suggest that differences of immune recovery between CBT and UBMT patients may exist even in patients surviving for more than 2 years and might be related to the development of chronic GVHD.

## Introduction

 Allogeneic hematopoietic cell transplantation (allo-HCT) has improved the prognosis of the patients with hematologic malignancies. Advances of conditioning regimens and immunosuppressive therapy contribute to the improvement of the prognosis. In addition, the introduction of unrelated bone marrow transplantation (UBMT) and cord blood transplantation (CBT) increased the chance to receive allo-HCT. These progresses have led to the result that some patients survive for more than a decade. Concomitantly, there have increased various problems to disturb the quality of life of long-term survivors after allo-HCT^1^^, ^^2^.

Chronic graft-versus-host disease (GVHD) is one of major problems for long-term survivors after allo-HCT, leading to reduced patient-reported quality of life and non-relapse mortality^3^^-^^5^.　 Risk factors for chronic GVHD include prior acute GVHD, donor peripheral blood stem-cell grafts, HLA disparity, female donors for male recipients, and recipient age^6^.　 Concerning the graft of allo-HCT, the incidence and severity of chronic GVHD are recently reported to be lower in CBT than UBMT patients^7^.

Acute GVHD is thought to be mediated primarily by mature donor T cells in the allogeneic stem cell product. By contrast, chronic GVHD is now considered to be more complex immune reaction. Both donor-derived effector T and B cells contribute to the pathology of chronic GVHD^8^^, ^^9^. In addition, regulatory elements within T and B cell lineages play important roles in the development and maintenance of immune tolerance after allo-HCT^10^^, ^^11^. Several reports have shown that the differences in immune reconstitution exist between the patients who received cord blood and other hematopoietic stem cell sources. In CBT patients, delayed recovery of T cells has been reported, by contrast, B cell numbers were higher compared to the patients received HLA-matched sibling or unrelated peripheral blood stem cell transplantation^12^^-^^14^. However, the observation duration was up to 2 years post allo-HCT. The differences in immune reconstitution more than 2 years after allo-HCT between CBT and UBMT have not been elucidated. 

In this study, we investigated the differences of immune reconstitution between CBT and UBMT patients, who survive for more than 2 years after allo-HCT without relapse of underlying disease, in relation to the development of chronic GVHD in our institute.

## MATERIALS AND METHODS

**Study design**

To determine whether the differences in immune reconstitution would exist between CBT and UBMT patients in long-survivors, we selected patients who had attended to our outpatient clinic for more than 2 years after allo-HCT and showed no symptoms of infections and relapse of underlying disease. Twenty-one patients who had received CBT (CBT group) and 20 patients who had received HLA-matched UBMT (UBMT group) from January 2002 to January 2014 were enrolled in this study. We collected peripheral blood for flow cytometric analysis to investigate immune reconstitution and clinical symptoms regarding allo-HCT at the time after allo-HCT described in duration in Table 1 after informed consent was given. We examined whether the differences in the immune reconstitution between CBT and UBMT patients who survive for more than 2 years after allo-HCT and any relations to the development of cGVHD could exist. This study was approved by the ethical committee of our institute.

**Patient characteristics**

Patient characteristics concerning allo-HCT are listed in Table 1. Median age in CBT group (45 years old, range: 24-63) was significantly older than in UBMT group (38 years old, range: 20-56) (p<0.01). But there was no significant difference in median duration from allo-HCT to the time, when the investigation was performed, between CBT (5.5 years, range: 2.4-13.1) and UBMT group (7.3 years, range: 2.1-14.1). Most of underlying disease were hematologic malignancies, only one patient in UBMT group was non-malignant disease (paroxysmal nocturnal hematuria/ aplastic anemia).

All of CBT were performed using cord blood which matched a minimum of 4 of 6 HLA loci to the patient at antigen level for HLA-A, HLA-B, and HLA-DRB1. In UBMT group, all patients received bone marrow but not peripheral blood stem cell from HLA-matched unrelated donor.

In 9 patients in CBT group and 12 patients in UBMT group, cyclophosphamide (60mg/kg x2) and total body irradiation (TBI) (3Gy x4) had been performed as conditioning regimen (myeloablative conditioning; MAC) (Table 1). In 12 patients in CBT group and 8 patients in UBMT group, various reduced intensity conditioning (RIC) had been conducted (Table 1). For the prophylaxis of GVHD, calcineurine inhibitor plus short-term methotrexate had been introduced into all of patients. 

**Table 1:** Clinical data in the patients who had received cord blood transplantation (CBT) and unrelated bone marrow transplantation (UBMT)

CBT								
Disease	Disease status	Age at allo-HCT	Gender	Source	CMV status	Conditioning	GVHD prophylaxis	Duration (years post allo-HCT)
AML	NC	57	F	CB	+	FLU+Mel+CA+TBI	CsA+sMTX	2.8
ATLL	CR1	57	M	CB	+	FLU+Mel+TBI	CsA+sMTX	3.4
Ph+ALL	CR2	48	M	CB	+	FLU+BU4+TBI	CsA+sMTX	2.4
DLBCL	CR2	51	F	CB	+	FLU+Mel+TBI	CsA+sMTX	2.4
CMMoL	NC	40	M	CB	+	CY+TBI	CsA+sMTX	3.9
Ph+ALL	CR2	44	M	CB	+	CY+TBI	CsA+sMTX	6.1
FL	NC	56	M	CB	+	FLU+Mel+TBI	CsA+sMTX	7.9
ALL	CR1	32	F	CB	+	CY+TBI	CsA+sMTX	9.5
FL	CR3	51	M	CB	+	FLU+Mel+TBI	CsA+sMTX	5.5
ALL	CR1	41	M	CB	+	FLU+Mel+TBI	CsA+sMTX	4.0
AML	CR1	45	M	CB	+	CY+AraC+TBI	CsA+sMTX	7.3
AML	CR2	45	F	CB	+	FLU+BU+TBI	CsA+sMTX	3.0
Ph+ALL	CR1	39	F	CB	-	CY+TBI	CsA+sMTX	10.8
AML	CR2	44	F	CB	+	CY+TBI	CsA+sMTX	7.1
PTCL	NC	33	M	CB	+	CY+TBI	CsA+sMTX	7.6
AML	CR1	52	F	CB	+	FLU+Mel+TBI	CsA+sMTX	3.5
AML	CR1	38	F	CB	+	CY+TBI	CsA+sMTX	2.1
CML	CP1	24	M	CB	+	CY+TBI	CsA+sMTX	13.1
ATLL	CR1	41	F	CB	+	FLU+Mel+TBI	CsA+sMTX	3.1
MDS	RA	62	M	CB	+	FLU+CA+Mel+TBI	CsA+sMTX	6.3
Ph+ALL	CR1	63	M	CB	+	FLU+Mel+TBI	CsA+sMTX	6.2
UBMT								
AML	CR1	25	F	uBM	+	CY+TBI	CsA+sMTX	8.8
MDS	RAEB	54	M	uBM	+	CY+TBI	CsA+sMTX	7.8
AML	CR1	34	M	uBM	+	CY+TBI	CsA+sMTX	3.4
ALCL	CR2	56	M	uBM	+	FLU+Mel+TBI	CsA+sMTX	4.3
AML	CR2	48	F	uBM	-	FLU+BU4+TBI	CsA+sMTX	5.0
MM	sCR	39	F	uBM	-	FLU+Mel+TBI	CsA+sMTX	2.1
DLBCL	CR2	44	F	uBM	+	FLU+BU2	CsA+sMTX	6.8
FL	CR2	46	F	uBM	+	FLU+Mel	CsA+sMTX	9.3
MDS	RAEB2	26	M	uBM	-	CY+TBI	CsA+sMTX	10.3
AML	CR1	26	F	uBM	+	CY+TBI	CsA+sMTX	13.6
MDS	RAEB	38	M	uBM	+	CY+TBI	CsA+sMTX	6.2
AML	CR1	38	F	uBM	+	CY+TBI	CsA+sMTX	8.0
PNH/AA		39	F	uBM	+	CY+FLU+ATG	CsA+sMTX	3.8
AML	CR1	49	M	uBM	+	CY+TBI	CsA+sMTX	10.2
MDS	RAEB2	29	M	uBM	+	CY+TBI	CsA+sMTX	8.0
CML	BC	35	F	uBM	+	CY+TBI	CsA+sMTX	11.7
NHL	CR2	32	M	uBM	+	CY+TBI	CsA+sMTX	14.1
CML	CR2	38	M	uBM	+	CY+TBI	CsA+sMTX	6.6
AML	CR2	20	F	uBM	+	FLU+BU2+TBI	Tac+sMTX	2.2
DLBCL	NC	48	F	uBM	+	FLU+BU2+TBI	CsA+sMTX	2.2

Allo-HCT; allogeneic hematopoietic cell transplantation, CMV; cytomegalovirus, GVHD; graft-versus-host disease, AML; acute myelogenous leukemia, ATLL; adult T cell leukemia/lymphoma, Ph+ALL; Philadelphia chromosome positive acute lymphoblastic leukemia, DLBCL; diffuse large B cell lymphoma, CMMoL; chronic myelomomocytic leukemia, FL; follicular lymphoma, ALL; acute lymphoblastic leukemia, PTCL; peripheral T cell lymphoma, CML; chronic myelogenous leukemia, MDS; myelodysplastic syndrome, ALCL; anaplastic large cell lymphoma, MM; multiple myeloma, PNH/AA; paroxysmal nocturnal hematuria/aplastic anemia, NHL; non-Hodgkin lymphoma, NC; non-complete remission, CR1; first complete remission, CR2; second complete remission, CR3; third complete remission, RA; refractory anemia, RAEB; refractory anemia with excess blast, sCR; stringent complete remission, BC; blastic crisis, CB; cord blood, UBMT; unrelated bone marrow transplantation, FLU; fludarabine, Mel; melphalan, CA; cytosine-arabinocyde, TBI; total body irradiation, BU; buslfan, CY; cyclophosphamide, CsA; cyclosporine A, sMTX; short-term methotrexate.

**Diagnosis of acute and chronic GVHD**

We collected clinical data from medical charts and diagnosed acute and chronic GVHD according to the previously described criteria^15^. 

**Flow cytometry**

Aliquots of whole peripheral blood were stained with various mAbs according to the manufacturer’s instructions. We had performed three-color flow cytometric analysis. mAbs used in this study were as follows: PC5-conjugated anti-CD4 mAb, PC5-conjugated anti-CD8 mAb, PE-conjugated anti-CD45RO mAb, FITC-conjugated anti-CD25 mAb, PE-conjugated anti-CD127 mAb, PC5-conjugated anti-CD19 mAb purchased from Beckman Coulter. FITC-conjugated anti-CD27 mAb and PE-conjugated anti-IgM mAb were purchased from Pharmingen. 

**Figure 1:**
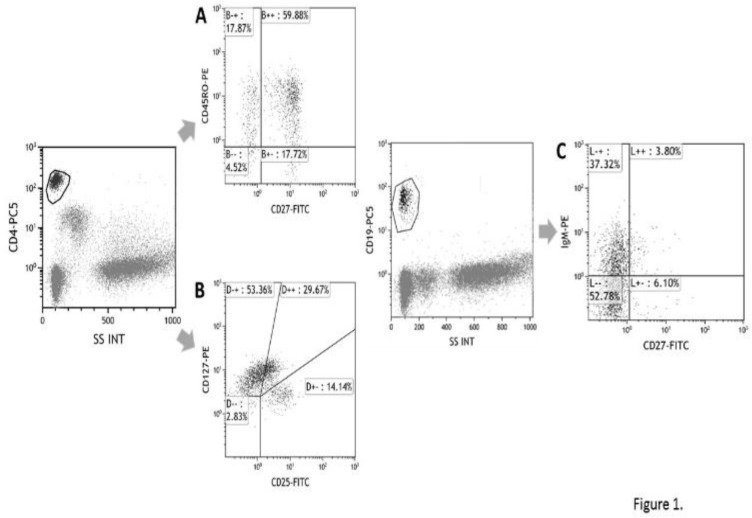
Representative data of flow cytometric analysis of the peripheral blood

In CD4+ or CD8+ cells, CD27+CD45RO- cells have been designated as naive T cell (Figure 1A), and CD25+CD127-~+- cells have been designated as regulatory T cell^16^ (Figure 1B). In CD19+ cells, CD27- cells have been designated as naive B cell and CD27+ B cells as memory B cell (Figure 1C). CD3-CD56+ cells have been determined as NK cell.

After gating CD4+ cells, we investigated the expression of CD45RO and CD27 (A). Similar analysis had been done in CD8+ cells. In addition, CD25+CD127negatvie to weekly positive cells in CD4+ cells were detected as regulatory T cells (B). In CD19+ cells, we investigated the expression of CD27 and IgM (C).

**Statistical analysis**

Immunologic parameters, described in Flow cytometry, were compared between the patients who received CBT and UBMT. In some investigation, immunologic parameters of the patients without versus with chronic GVHD were compared. A comparison between the two groups was performed using Mann-Whitney U test. 

## Results

**Reconstitution in T cell between CBT and UBMT group**

**Figure 2:**
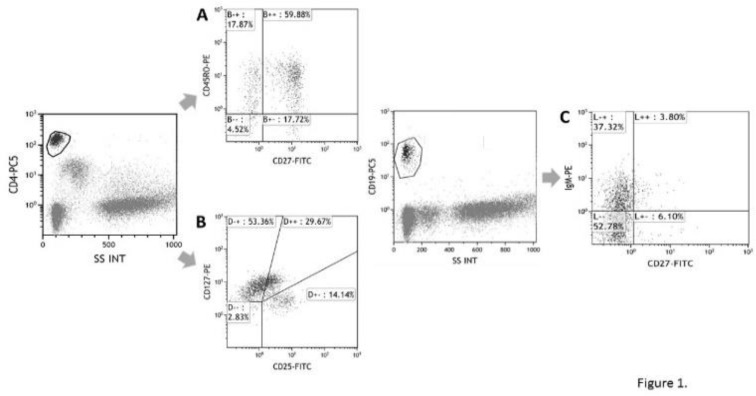
Differences in T cell recovery between CBT and UBMT group Absolute count of CD4+ cell (A), CD8+ cell (B), CD4/CD8 ratio (C), and regulatory T cell (D) of peripheral blood were compared between CBT and UBMT group. * indicates differences that are statistically significant (p<0.05).

We first examined the differences in the reconstitution of T cells between CBT and UBMT group. As shown in Figure 2A, there was no significant difference in absolute number of CD4+ T cell, but significant differences were observed in absolute number of CD8+ cell and CD4/CD8 ratio between the two groups. Absolute cell number of CD8+ cell was significantly higher in UBMT patients (Figure 2B). As a result, CD4/CD8 ration was significantly lower in UBMT patients (Figure 2C). We next examined the ratio and cell count of subpopulations in T cells. In CD4+ and CD8+ cells, no significant difference in ratio and absolute cell number of naive cell (CD27+CD45RO-) was observed between the two groups as well as memory cell (CD27-CD45RO+) (data not shown). Furthermore, we have observed no significant difference in absolute cell number of regulatory T cell, which has been reported to be involved in the pathogenesis of chronic GVHD (Figure 2D). 

**Reconstitution in B cell and NK cell**

**Figure 3:**
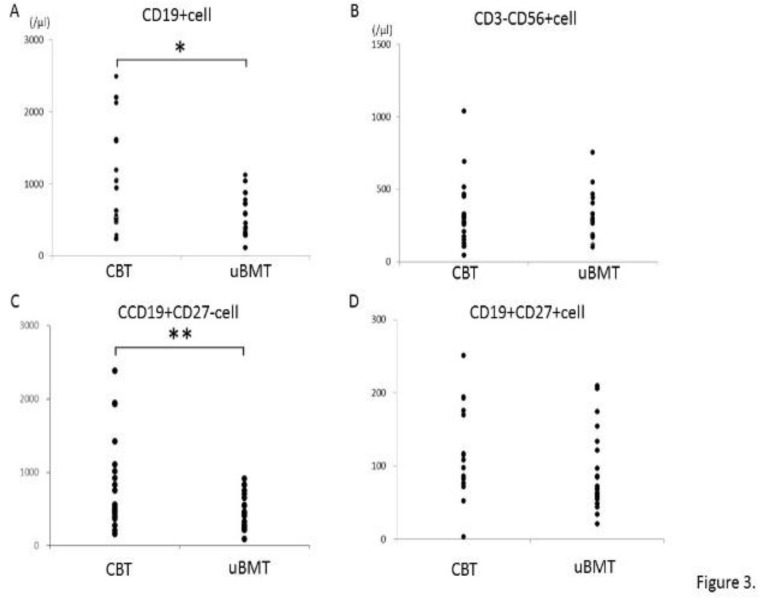
Differences in B cell recovery between CBT and UBMT group

We next examined the recovery of B cells. As shown in Figure 3A, absolute cell number of CD19+ cell in UBMT group was significantly lower than in CBT group (p<0.05), although there was no significant difference in serum levels of immunoglobulins (IgG, IgA, and IgM) (data not shown). By contrast, no significant difference was observed in NK cell counts between the two groups (Figure 3B). Absolute cell number of naive B cell (CD19+CD27-) was also lower in UBMT group without statistical significance (P=0.05) (Figure 3C). In memory B cells (CD19+CD27+), absolute cell number tended to be lower in UBMT than CBT group without statistical significance (Figure 3D, p=0.10). These results suggest that the differences in the reconstitution of B cells between CBT and UBMT patients at a long-term period after allo-HCT could exist.

**Complications after allo-HCT including chronic GVHD in CBT and UBMT group**

Complications after allo-HCT have been summarized in Table 2. Acute GVHD (grade II-IV) was observed in 7 and 6 patients in CBT and UBMT groups, respectively. CMV antigenemia was more frequently detected in CBT group but cleared in all of cases within 6 months after allo-HCT. In addition, bacterial infections such as sepsis and febrile neutropenia were more frequently complicated in CBT group, as described previously^17^^, ^^18^. Chronic GVHD was observed in 7 of 21 patients in CBT group and 8 of 20 patients in UBMT group. Three of 7 patients in CBT group and 5 of 8 patients in UBMT group were diagnosed as extended type of chronic GVHD. Continuation of immnosuppressive therapy has been required in 2 and 3 patients in CBT and UBMT group, respectively. We have found no clear difference in the frequency and the severity of chronic GVHD between the two groups in our institute, although the incidence of chronic GVHD has been reported to be higher in UBMT than CBT patients^7^. 

To investigate whether the differences of immune reconstitution would exist between CBT and UBMT group in relation to the development of chronic GVHD, we further divided the patients into 4 groups according to the presence or absence of chronic GVHD as follows: the patients with cGVHD in CBT group (n=7), without cGVHD in CBT group (n=14), without cGVHD in UBMT group (n=12), with cGVHD in UBMT group (n=8).

**Immune reconstitution in T and B cells in relation to chronic GVHD**

**Figure 4:**
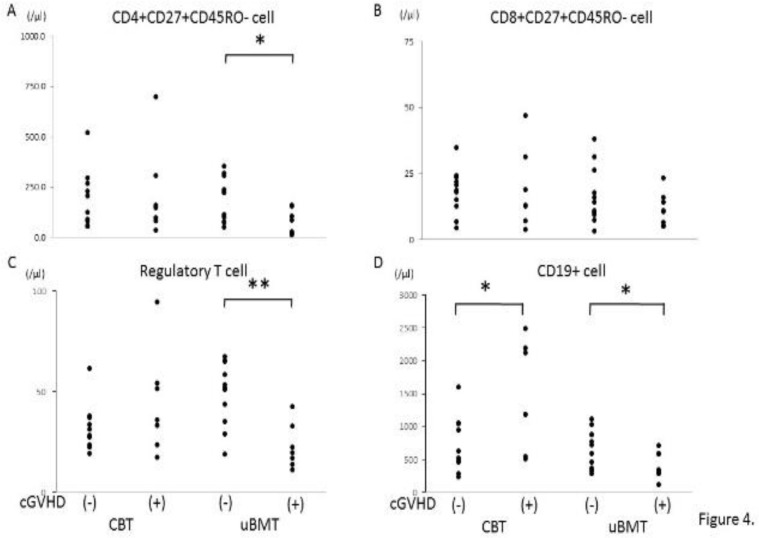
Comparison of immune recovery in T and B cells between the patients with and without chronic GVHD

We examined T cell recovery between 4 groups described above. There was no significant difference in CD4+ and CD8+ cell number and CD4/CD8 ratio between 4 groups (data not shown). As shown in Figure 4A and B, absolute cell number in peripheral blood of naive CD4+ but not naive CD8+ T cells was significantly lower in the patients with cGVHD in UBMT group. Absolute cell number of regulatory T cell was lower in the patients with cGVHD in UBMT group without statistical significance (p=0.08, Figure 4C).

In B cells, absolute cell number of CD19+ cell in peripheral blood was higher in the patients with cGVHD in CBT group, but conversely lower in the patients with cGVHD in UBMT group, as shown in Figure 4D.

These results suggest that there exist some differences in immune reconstitution of both T and B cells in relation to the development of chronic GVHD between CBT and UBMT group

Absolute cell count of naïve CD4+ cell (A), naïve CD8+ cell (B), regulatory T cell (C), and CD19+ cell (D) of peripheral blood were compared between the patients with and without chronic GVHD in CBT and UBMT group. * indicates differences that are statistically significant (p<0.05). ** indicates p=0.08.

**B cell subpopulations in relation to chronic GVHD among CBT and UBMT group**

To more precisely investigate the differences in B cell reconstitution between the two groups in relation to chronic GVHD, we next examined the subpopulation of B cells.

**Figure 5:**
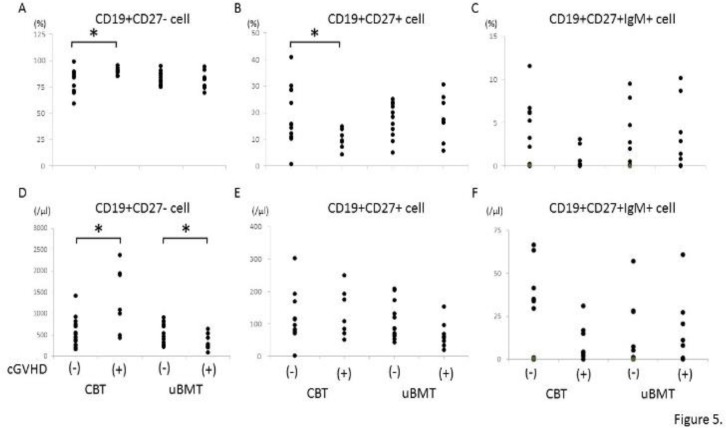
Comparison of immune recovery in B cell subpopulations between the patients with and without chronic GVHD

Both ratio and absolute cell number of naive B cell, which express CD19 but not CD27, were significantly higher in the patients with than without cGVHD in CBT group (Figure 5A and D). By contrast, absolute cell number but not ratio of naive B cell was significantly lower in the patients with than without cGVHD in UBMT group (Figure 5A and D). Concerning memory B cells, ratio but not absolute cell number of CD19+CD27+ cell in peripheral blood was significantly lower in the patients with than without cGVHD in CBT group, while there was no significant difference in memory B cells between the patients with and without chronic GVHD in UBMT group (Figure 5B and E). 

Regulatory B cells, which are also suggested to be involved in the pathogenesis of chronic GVHD, have been reported to be enriched in the IgM memory and transitional subsets^11^^, ^^19^. Therefore, we next examined the IgM memory B cells, designated as the cells which express CD19, CD27, and IgM on cell surface. As shown in Figure 5C and F, both ratio and absolute cell count were lower in the patients with cGVHD in CBT group, but the differences were not statistically significant. In UBMT group, we observed no significant difference in IgM memory B cells between the patients with and without chronic GVHD.

These results suggest that different immune reconstitution in T and B cells, which would be related to the development of chronic GVHD, could exist between CBT and UBMT patients. 

The ratio of naïve B cell (A), memory B cell (B), and IgM memory B cell (C) of peripheral blood were compared between the patients with and without chronic GVHD in CBT and UBMT group (upper column). Absolute cell number of naïve B cell (D), memory B cell (E), and IgM memory B cell (F) of peripheral blood were compared between the patients with and without chronic GVHD in CBT and UBMT group (lower column). * indicates differences that are statistically significant (p<0.05).

**Table 2:** Complications after allo-HCT in the patients who had received cord blood transplantation (CBT) and unrelated bone marrow transplantation (UBMT)

CBT								
Disease	Disease status	Age at allo-HCT	Gender	aGVHD (grade)	infection	CMV antigenemia	cGVHD	Sites of cGVHD
AML	NC	57	F	I	-	+	L	skin
ATL	CR1	57	M	II	sepsis	+	L	skin
Ph+ALL	CR2	48	M	I	FN	+	L	mouth
DLBCL	CR2	51	F	I	sepsis	+	E	lung
CMMoL	NC	40	M	II	Tbc	+	E	skin, liver
Ph+ALL	CR2	44	M	0	FN	+	-	
FL	NC	56	M	0	FN	+	E	lung
ALL	CR1	32	F	I	-	+	-	
FL	CR3	51	M	0	FN	+	L	
ALL	CR1	41	M	0	sepsis	+	-	
AML	CR1	45	M	0	-	+	-	
AML	CR2	45	F	III	sepsis	-	-	
Ph+ALL	CR1	39	F	II	sepsis	+	-	
AML	CR2	44	F	II	sepsis	+	-	
PTCL	NC	33	M	II	sepsis	+	-	
AML	CR1	52	F	0	FN	+	-	
AML	CR1	38	F	II	-	+	-	
CML	CP1	24	M	0	FN	+	-	
ATL	CR1	41	F	0	FN	+	-	
MDS	RA	62	M	I	hemorrhagic cystitis	+	-	
Ph+ALL	CR1	63	M	I	-	+	-	
UBMT								
Disease	Disease status	Age at allo-HCT	Gender	aGVHD (grade)	infection	CMV antigenemia	cGVHD	Sites of cGVHD
AML	CR1	25	F	0	-	+	-	
MDS	RAEB2	54	M	I	-	+	L	mouth
AML	CR1	34	M	II	FN	+	E	lung
ALCL	CR2	56	M	II	sepsis	+	E	mouth, eye
AML	CR2	48	F	0	sepsis	-	-	
MM	sCR	39	F	I	FN	-	-	
DLBCL	CR2	44	F	0	-	-	-	
FL	CR2	46	F	I	pneumonia	-	E	eye, lung
MDS	RAEB2	26	M	0	FN	-	-	
AML	CR1	26	F	I	-	+	-	
MDS	RAEB2	38	M	0	FN	-	-	
AML	CR1	38	F	I	-	+	-	
PNH/AA		39	F	0	pneumonia	+	-	
AML	CR1	49	M	I	-	+	-	
MDS	RAEB2	29	M	III	hemorrhagic cystitis	+	L	skin
CML	BC	35	F	I	FN	+	E	eye, skin, lung
NHL	CR2	32	M	I	FN	+	-	
CML	CR2	38	M	III	-	+	E	skin, mouth, eye
AML	CR2	20	F	I	-	-	L	skin, eye
DLBCL	NC	48	F	III	FN	+	-	

Allo-HCT; allogeneic hematopoietic cell transplantation, CMV; cytomegalovirus, GVHD; graft-versus-host disease, AML; acute myelogenous leukemia, ATLL; adult T cell leukemia/lymphoma, Ph+ALL; Philadelphia chromosome positive acute lymphoblastic leukemia, DLBCL; diffuse large B cell lymphoma, CMMoL; chronic myelomomocytic leukemia, FL; follicular lymphoma, ALL; acute lymphoblastic leukemia, PTCL; peripheral T cell lymphoma, CML; chronic myelogenous leukemia, MDS; myelodysplastic syndrome, ALCL; anaplastic large cell lymphoma, MM; multiple myeloma, PNH/AA; paroxysmal nocturnal hematuria/aplastic anemia, NHL; non-Hodgkin lymphoma, NC; non-complete remission, CR1; first complete remission, CR2; second complete remission, CR3; third complete remission, RA; refractory anemia, RAEB; refractory anemia with excess blast, sCR; stringent complete remission, BC; blastic crisis, CB; cord blood, UBMT; unrelated bone marrow transplantation, FN; febrile neutropenia, Tbc; tuberculosis, L; limited type, E; extensive type.

## Discussion

 Recent advances of allo-HCT improve the prognosis of the patients with hematopoietic malignancies and result in the long-term survivors, while there have appeared several problems such as chronic GVHD in long-term survivors. Development of chronic GVHD results in the decrease of quality of life and increase non-relapse mortality in long-term survivors^3^^-^^5^. Recently, the pathogenesis of chronic GVHD has been elucidated^8^^, ^^9^. In the development of chronic GVHD, effector cells as well as regulatory cells have been reported to be involved^10^^, ^^11^^, ^^19^. Several reports have shown the differences in immune recovery between the patients transplanted with cord blood and peripheral blood stem cells from HLA-matched sibling or unrelated donor^12^^-^^14^. However, observation period for immune recovery was up to 2 years in these reports. It has been elucidated whether the differences of immune reconstitution between CBT and UBMT patients surviving more than 2 years would exist and relate to the development of chronic GVHD, although the incidence and the severity of chronic GVHD have been reported to be lower in CBT than UBMT patients among Japanese patients^7^.

In this study, we investigated the immunologic reconstitution by flow cytometric analysis in the patients who received CBT or UBMT, although the duration from allo-HCT to investigating time was varied in each patient. We observed several differences in both T- and B-cell lineages between the patients who received CBT and UBMT. Absolute cell number of CD8+ T cell was significantly higher in UBMT group, while no difference was observed in CD4+ T cell. As a result, the ratio of CD4/CD8 was significantly lower in UBMT group (Figure 2). In respect to B cells, absolute cell number of B cell was significantly lower in UBMT group (Figure 3A). In addition, absolute cell number of naive B cell was lower in UBMT group, but not significantly (Figure 3C, p=0.05). These results suggest that different immune reconstitution between CBT and UBMT patients could exist even more than 2 years after allo-HCT. 

Nine of 21 patients in CBT group and 12 of 20 patients in UBMT group had received MAC as conditioning regimen. The difference of conditioning regimen (MAC versus RIC) would possibly affect the immune reconstitution in the patients surviving more than 2 years after allo-HCT. However, Wike et al. investigated the effect of conditioning regimen on marrow damage and hematopoietic recovery in adult leukemia patients. They reported that MAC regimen including TBI leads to prolonged reduction in marrow cellularity, but does not show additional histological long-term injury and differences in immunologic recovery^20^. In addition, Geyer et al. also showed that immune cell subset recovery, immunoglobulin reconstitution, and the incidence of opportunistic infections did not differ significantly between MAC and RIC regimen in pediatric CBT cohort^21^. These results can suggest that differences of conditioning regimen would not affect immune reconstitution in our investigation. 

Regarding the relation to the occurrence of chronic GVHD, several differences have found between the patients with and without chronic GVHD in both CBT and UBMT group.

In respect to T cells, absolute cell number of naive CD4+ T cell was significantly lower in the patients with chronic GVHD in UBMT group (Figure 4A), while no significant difference was observed in naive CD8+ T cells and regulatory T cells (Figure 4B and C). Although regulatory T cells have been reported to be involved in the pathogenesis in chronic GVHD^10^, we have no significant difference between the patients with and without chronic GVHD in both groups. Although it has been observed that absolute cell number of regulatory T cell was lower in the patients with chronic GVHD in UBMT group, the difference was not statistically significant (p=0.08) (Figure 4C). Immune reconstitution of T cells may be different between CBT and UBMT patients even in long-term survivors and might be related to the pathogenesis of chronic GVHD at least in UBMT patients. 

Concerning B cells, significant differences of absolute cell number of CD19+ B cell between the patients with and without chronic GVHD were observed in both CBT and UBMT group (Figure 4D). In addition, absolute cell number of naive B cell significantly differed between the patients with and without chronic GVHD in both groups. Absolute cell number of naive B cell was significantly higher in the patients with chronic GVHD in CBT group, while significantly lower in UBMT group (Figure 5D). As we could not examine regulatory B cells in this study, we compared IgM memory B cells, which have been reported to contain regulatory B cells^11^, between CBT and UBMT patients in relation to the development of chronic GVHD. There was no significant difference between the patients with and without chronic GVHD in both groups, although IgM memory B cells were lower in the patients with chronic GVHD in CBT group, but not statistically significant. However, our results suggest that immune reconstitutions of B cells play important roles in the pathogenesis of chronic GVHD in both CBT and UBMT patients，being consistent to previous reports^22^^, ^^23^. 

Taken together, our results suggest that the differences in immune reconstitution of T and B cells may exist between CBT and UBMT patients even in long-term survivors and might be related to the differences in the development of chronic GVHD between CBT and UBMT patients. 

Treatment for chronic GVHD has been still difficult and not satisfactory, but new strategies for the treatment of chronic GVHD have been recently evolved^24^^-^^26^. Low-dose infusion of interleukin-2 to stimulate regulatory T cells has been reported to improve symptoms of chronic GVHD^27^. Infusion of rituximab targeting for B cells has also been reported to be effective to chronic GVHD^26^^, ^^28^. Investigating the immunological differences in the development of chronic GVHD between CBT and UBMT would provide the understanding of the pathogenesis of chronic GVHD and the clue to introduce more effective treatment modalities for chronic GVHD. 

In this study, there is no significant difference in the observation period from allo-HCT between two groups, although age at allo-HCT was older in CBT group. Moreover, in all of patients, GVHD prophylaxis was calcineurine inhibitor and short-term methotrexate. 

## CONCLUSION

 Our results showed that the differences of immune reconstitution may exist between CBT and UBMT patients even in long- term survivors and might be correlated to the development of chronic GVHD, regardless of the small number of patients examined and the fact that longitudinal analysis was not used in our study. Studies in larger scale and more precise examinations of T and B cells would more clearly reveal the differences in the immune reconstitution and the roles of the pathogenesis of chronic GVHD in CBT and UBMT patients.
